# Multipoint surface electromyography measurement using bull’s-eye electrodes for wide-area topographic analysis

**DOI:** 10.1186/s40101-023-00342-3

**Published:** 2023-10-27

**Authors:** Megumi Shimura, Akihiko Mizumoto, Yali Xia, Yoshihiro Shimomura

**Affiliations:** 1 Graduate School of Science and Engineering, Chiba University, 1-33 Yayoi-Cho, Inage-Ku, Chiba City, 2638522 Japan; 2 Design Research Institute, Chiba University, 1-33 Yayoi-Cho, Inage-Ku, Chiba City, 2638522 Japan

**Keywords:** Multipoint surface electromyography, Entropy, Muscle groups, Measurement method

## Abstract

**Background:**

Surface electromyography (sEMG) is primarily used to analyze individual and neighboring muscle activity. However, using a broader approach can enable simultaneous measurement of multiple muscles, which is essential for understanding muscular coordination. Using the “bull’s-eye electrode,” which allows bipolar derivation without directional dependence, enables wide-area multipoint sEMG measurements. This study aims to establish a multipoint measurement system and demonstrate its effectiveness and evaluates forearm fatigue and created topographic maps during a grasping task.

**Methods:**

Nine healthy adults with no recent arm injuries or illnesses participated in this study. They performed grasping tasks using their dominant hand, while bull’s-eye electrodes recorded their muscle activity. To validate the effectiveness of the system, we calculated the root mean squares of muscle activity and entropy, an indicator of muscle activity distribution, and compared them over time.

**Results:**

The entropy analysis demonstrated a significant time-course effect with increased entropy over time, suggesting increased forearm muscle uniformity, which is possibly indicative of fatigue. Topographic maps visually displayed muscle activity, revealing notable intersubject variations.

**Discussion:**

Bull’s-eye electrodes facilitated the capture of nine homogeneous muscle activity points, enabling the creation of topographic images. The entropy increased progressively, suggesting an adaptive muscle coordination response to fatigue. Despite some limitations, such as inadequate measurement of the forearm muscles’ belly, the system is an unconventional measurement method.

**Conclusion:**

This study established a robust system for wide-area multipoint sEMG measurements using a bull’s-eye electrode setup. This system effectively evaluates muscle fatigue and provides a comprehensive topographic view of muscle activity. These results mark a significant step towards developing a future multichannel sEMG system with enhanced measurement points and improved wearability.

**Trial registration:**

This study was approved by the Ethics Committee of Chiba University Graduate School of Engineering (acceptance number: R4-12, Acceptance date: November 04, 2022).

## Background

Surface electromyography (sEMG) is a widely used non-invasive method for measuring the electrical activity of skeletal muscles, enabling muscle activity and fatigue assessment [1]. Despite various analysis techniques [2, 3], the conventional sEMG methodology remains unchanged. The two prevailing approaches include using a pair of electrodes on a specific muscle [4, 5] or employing a dense grid of electrodes arranged in a small rectangular layout [6–8]. However, these methods focused on individual or neighboring muscles and neglected the importance of simultaneously measuring multiple muscles, which is critical for understanding muscular coordination in body control [9].

Multipoint sEMG measures muscle activity through the unipolar derivation of each electrode [10] or bipolar derivation of adjacent electrodes [11, 12]. However, both methods encounter challenges when attempting a comprehensive measurement. Unipolar derivation, common in electroencephalogram (EEG) measurements [13, 14], faces issues with sEMG owing to motion-induced electrical potential changes and uncertain indifferent electrode locations, making it susceptible to spatial noise [15]. Even under noise-free conditions, forming equipotential lines within the measurement area is not feasible, and the low-pass filter effect of biological tissue is amplified by the electrode’s distance from the indifferent electrode, leading to varying frequency components requiring careful potential comparison.

In contrast, conventional bipolar derivation requires the alignment of the two electrodes in the myofiber direction, which becomes problematic at muscle boundaries or when assessing muscles with differing myofiber directions [16]. To address this, the bull’s-eye electrode [17, 18], designed for anisotropy-free bipolar derivation, employs a central pin electrode and a concentric ring electrode configuration. This design eliminates the need for electrode-muscle fiber alignment, conductive paste, or gel, making it suitable for wide-area multipoint sEMG measurements.

This study aimed to establish a multipoint measurement system without directionally dependent measurement points. Muscle fatigue was assessed topographically using grasping tasks involving multiple muscles, and the ability to detect significant changes in integrated indices in a time series was examined to demonstrate the effectiveness of the system.

## Methods

### Subjects

Nine healthy adults who did not sustain any injuries or illnesses in their dominant arm in the last 6 months participated (5 males and 4 females, aged 24 ± 2 years, body weight 60 ± 8 kg, height 167 ± 9 cm, and grip strength of dominant hand 271 ± 53 N). All the participants were right-handed according to the Edinburgh Handedness Test.

### Tasks

**Fig. 1:**
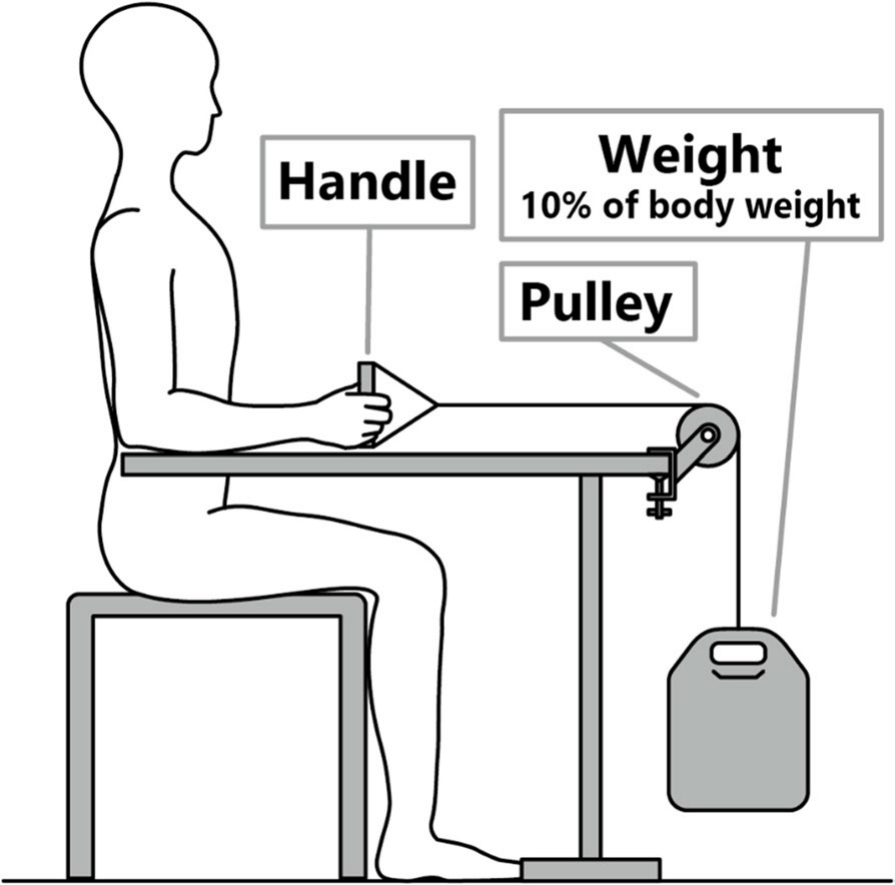
Subject’s posture and experimental apparatus

The participants sat on a chair with their dominant upper limb hanging by their sides, the elbow joint flexed at 90°, and the forearm resting on a desk. The forearm was in a neutral pronation, supination, flexion, and extension position, with the palm oriented perpendicular to the desk (Fig. 1). A pulley system was used with a handle attached to one end of the string and a weight equal to 10% of the subject’s body weight on the other, providing a constant external force. Subjects were instructed to grasp the handle with their dominant hand, maintain a constant weight height while keeping their forearm muscles engaged in a neutral position, and avoid lifting their arms off the desk. Three repetitions of the task were performed with rest periods between. Subsequently, the subjects exerted a maximum grip force for 5 s in the same posture, and this measurement was taken twice.

### Measurements

**Fig. 2:**
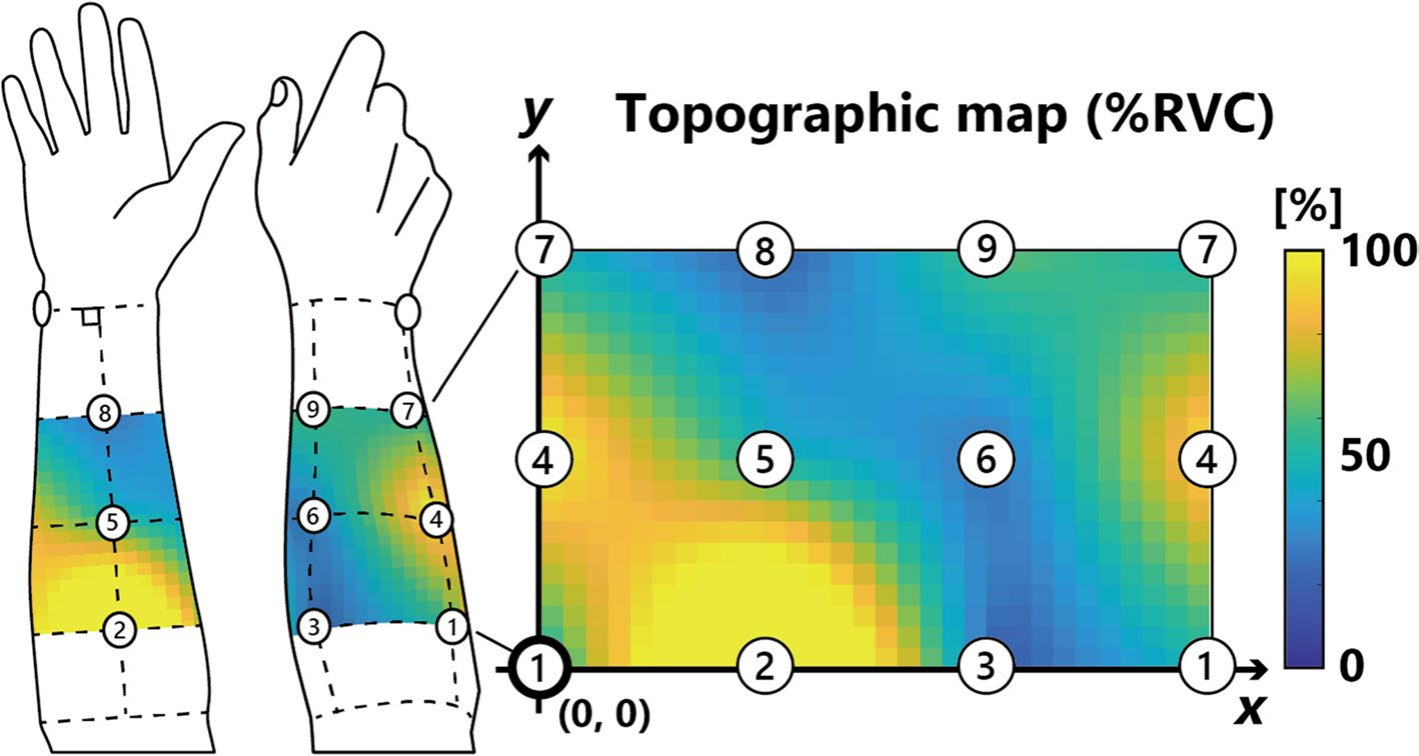
Topographic map and electrodes position. Nine bull’s-eye electrodes were placed on the right forearm in a three-by-three grid based on the length of the forearm (distance from the head to the styloid process of the radius). An Ag/AgCl ground electrode was placed on the ulnar styloid process. The %RVC was used to create topographic maps via interpolation to estimate the values between electrodes. The position of electrode 1 corresponds to the lower left coordinate (0,0) on the map

Bull’s-eye electrodes, which were made by the author, were used in the experiment. These electrodes consisted of two brass rings: one large (20 mm diameter and 15 mm inner diameter) and one small (7 mm diameter and 3 mm inner diameter), with a plate thickness of 0.5 mm. Nine bull’s-eye electrodes were evenly spaced on the subject’s forearm based on the forearm length and wrist circumference (Fig. 2). Before electrode placement, the skin was cleaned, and a conductive paste was applied. The potentials obtained from the electrodes were processed through a buffer amplifier, sampled at 2 kHz, amplified 2000 times, and filtered through a 5–500-Hz bandpass filter (MP150, BIOPAC System Inc.).

### Data analysis

The root mean square (RMS) was calculated every 0.5 s for each channel and during the central 3 s of maximum grip force. The average of two measurements was used as the reference voluntary contraction (RVC) [19]. The RMS values during the tasks were divided by the RVC to calculate the %RVC, which was used to generate topographic maps (Fig. 2) using MATLAB (MathWorks Inc.).

Entropy analysis was used to evaluate fatigue in muscle groups. Entropy measures the uncertainty or randomness associated with signals or data sources and is widely used in information theory [20]. In a previous study, entropy was used to study changes in trapezius muscle activity during fatigue tasks [8]. Higher entropy values indicate a more uniform distribution of muscle activity and changes in entropy are expected to occur with muscle group fatigue owing to alterations in active alternations and patterns. Entropy was calculated for each channel every 0.5 s as follows:

$$Entr= -\sum_{i=1}^{9}{p}^{2}\left(i\right){\mathrm{log}}_{2}{p}^{2}\left(i\right)$$

$${p}^{2}\left(i\right)=\frac{{RMS}_{i}}{\sum_{k=1}^{9}{RMS}_{k}}$$

The probability, *p*^*2*^*(i)*, which indicates the activity rate of one point relative to the total area of measurement, was obtained by dividing the RMS of electrode i by the sum of nine RMS values. To normalize the entropy, the average of each subject’s task was calculated and subtracted. The task segment was divided into six equal segments of 30 s each, and the average of each segment was calculated.

To analyze entropy, one-way repeated measures ANOVA and a multiple comparison test (Tukey–Kramer method) were performed, with the time course as a factor. The significance level was set to 5%. Eight participants were included in the statistical analyses. One subject was excluded from the analysis because some electrodes floated off the skin during measurement, resulting in missing data.

## Results

**Fig. 3:**
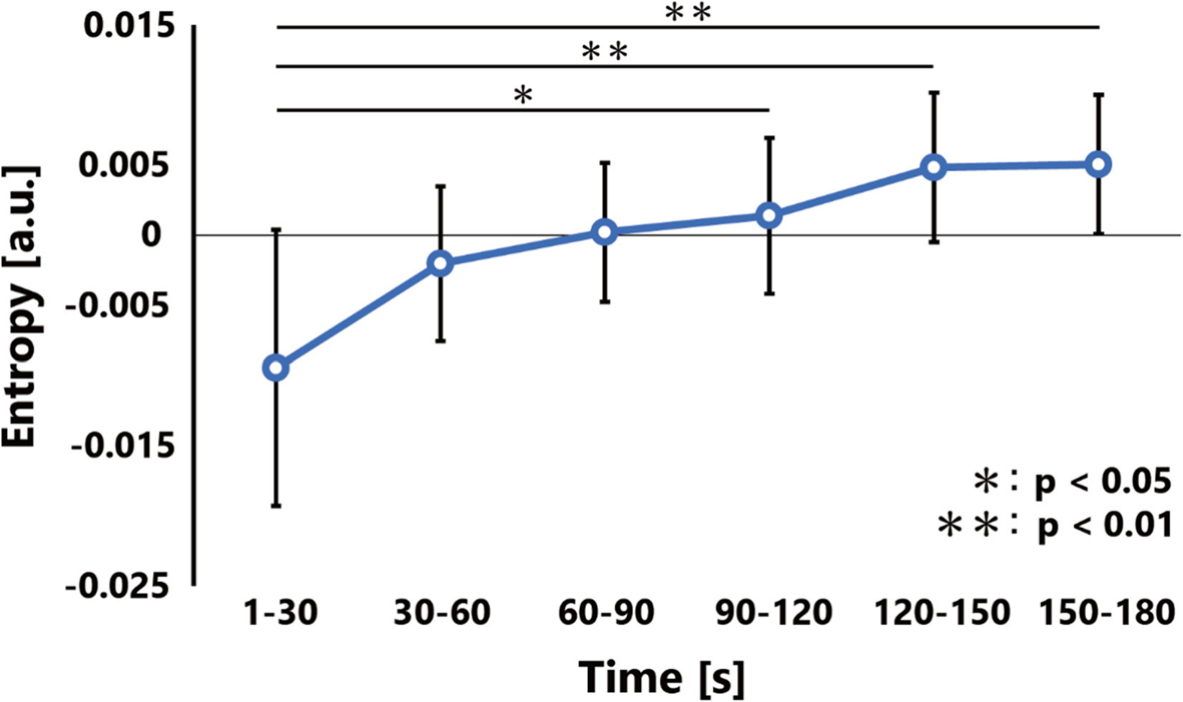
Entropy results. Shown as mean and standard deviation

**Fig. 4:**
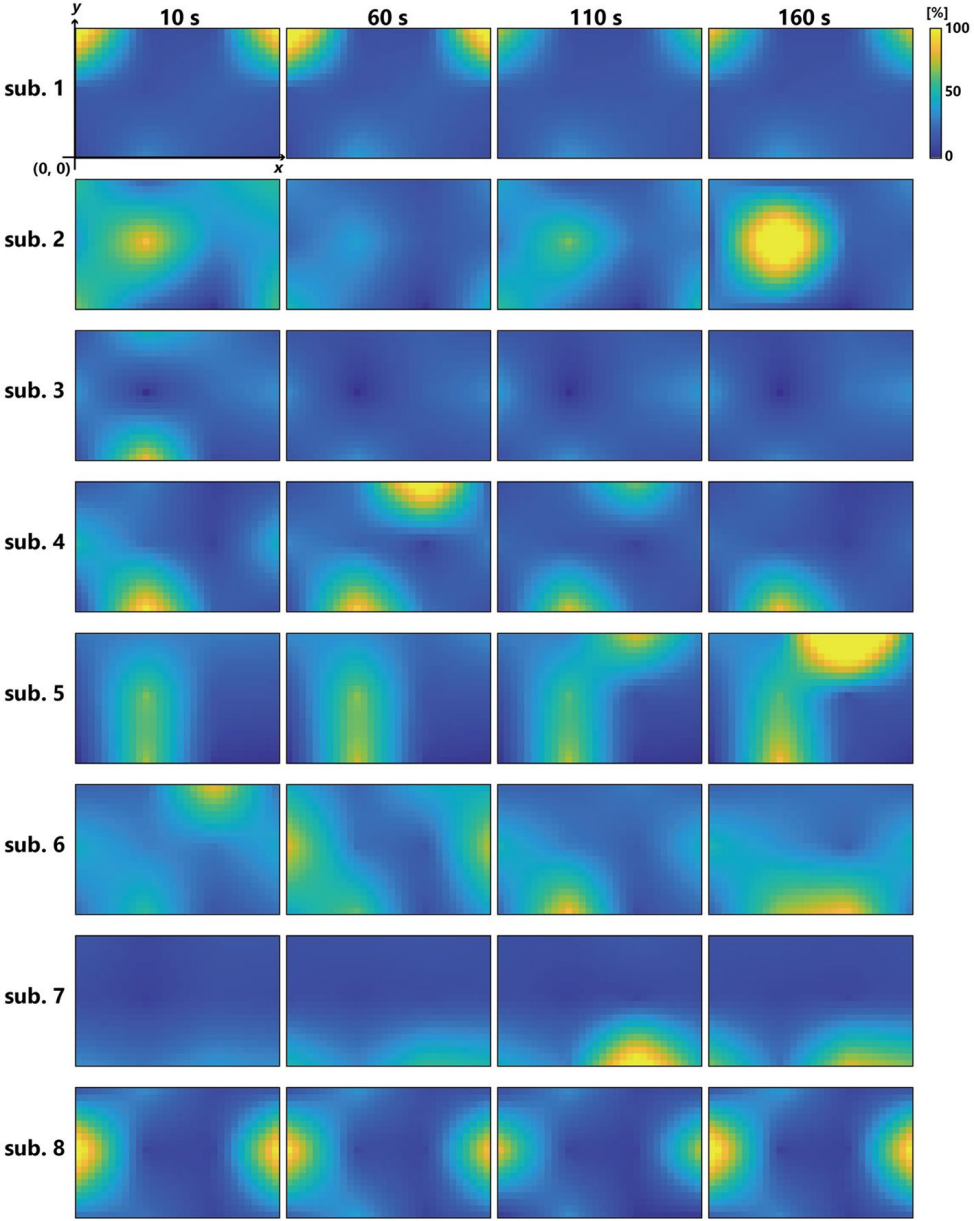
%RVC topographical maps. Topographic maps for eight subjects at 10, 60, 110, and 160 s after the start of the first trial are shown. The orientation of all topographic maps is the same as shown in Fig. 2

The results of the entropy analysis revealed a significant main effect for the time-course factor (*F* (5, 40) = 5.15, *p* < 0.001), with significant increases in the 90–120 s (*p* = 0.027), 120–150 s (*p* = 0.002), and 150–180 s (*p* = 0.001) segments compared to the 1–30 s segment. Additionally, there was a significant increase in the 120–150 s (*p* = 0.002) and 150–180 s (*p* = 0.001) segments (Fig. 3). Topographic maps of the eight subjects are shown in Fig. 4.

## Discussion

Bull’s-eye electrodes allowed for the measurement of nine homogeneous points, generating topographical images. This allowed the visualization of muscle activity and variations in activity. In this study, the electrode placement was defined based on two points on the radius. This ensured within-subject reproducibility of the measurement points. In addition, because the muscles were not located by palpation, the electrode application time was reduced compared to the standard bipolar recording method.

This study applied external force based on body weight rather than the maximum voluntary contraction (MVC) to enable the subjects to start the task without fatigue. In addition, grip strength and body weight are correlated in healthy young adults [21]. The external force was converted to %MVC, resulting in an average of 22.3 ± 4.8% MVC. Previous studies have revealed signs of fatigue in the extensors and flexors within 10 and 30 s, respectively, at 25% MVC [22], suggesting significant fatigue after 180 s of grasping.

Entropy exhibited a progressive increase over time, indicating enhanced forearm muscle activity uniformity at 150 s compared to that at the beginning of the task, potentially due to muscle fatigue. When muscle fatigue occurs, central fatigue reduces the firing frequency of the motor units, leading to alternating recruitment to compensate for the loss of force production [23]. This adaptive mechanism maintains performance by resting the motor units while activating others [24]. Such adaptations also occur at the muscle group level, where changes in muscle coordination (number and composition of active muscles) serve as strategies to counteract fatigue [25]. Turpin et al. reported that, when fatigue occurs during a cyclic task, flexible combinations of several muscle synergy groups maintain muscle activity levels [25]. In this study, although a simple static task, multiple muscle synergy groups in the forearm attempted to fire and maintain performance, which likely resulted in an increase in muscle activity uniformity in the latter half of the task. Additionally, Fig. 4 displays intersubject differences in muscle activity areas, implying that even in simple tasks, individual variations in fatigue coping strategies can be observed through extensive topographical descriptions.

A limitation of this study was the inadequate measurement of the belly of the forearm muscles because the electrodes were placed in 25–75% of the forearm. Challenges in stably placing the device near the elbow joint due to uneven muscle belly and elbow fossa led to noise generation. Future improvements to the device will enable measurements closer to the elbow joint. The positional relationship between the electrodes and muscles will be further investigated to ensure stable measurements.

## Conclusion

This study successfully established a system for measuring a wide range of multipoint sEMG using bull’s-eye electrodes, enabling bipolar derivation without directional dependence, and reducing the burden of electrode positioning. The system demonstrated its capability to evaluate fatigue using entropy and provided extensive topographical descriptions of muscle activity across a wide range. In the future, we aim to develop a multichannel sEMG system with more measurement points and improved wearability to assess broader areas of muscle activity.

## Data Availability

The datasets analyzed in the current study are available from the corresponding author upon request.

## Abbreviations

EEG: Electroencephalogram
RMS: Root mean square
RVC: Reference voluntary contraction
sEMG: Surface electromyography
MVC: Maximum voluntary contraction
